# In-hospital mortality after surgery: a retrospective cohort study in a Japanese university hospital

**DOI:** 10.1186/s40064-016-2279-1

**Published:** 2016-05-21

**Authors:** Yo Shidara, Yoshihisa Fujita, Saiko Fukunaga, Kae Ikeda, Mayumi Uemura

**Affiliations:** Department of Anesthesiology and ICM, Kawasaki Medical School, 577 Matsushima, Kurashiki, Okayama 7010192 Japan; Department of Anesthesiology, Iwaki Kyoritsu General Hospital, 16 Kusehara, Uchigo Mimaya-machi, Iwaki, Fukushima 973-8555 Japan

**Keywords:** In-hospital mortality, Surgery, Anesthesia, Quality of care

## Abstract

**Background:**

The rapidly aging population affects Japan’s health system, which is characterized by equity and full health insurance coverage for the entire population. However, the current outcomes after surgery in tertiary hospitals in Japan are not known. We aimed to gain an overview of postoperative mortality and death in a tertiary university hospital.

**Methods:**

Using the administrative database of Kawasaki Medical School Hospital, we investigated the pattern of in-hospital mortality and death for patients who underwent surgery under general or regional anesthesia between January 2010 and December 2011. We used a logistic regression model to find pre-operative risk factors associated with in-hospital mortality in this derivation cohort and tested its results in the validation cohort obtained from surgical patients between January 2012 and April 2014.

**Results:**

Among 8414 admissions for surgery patients aged ≥65 years was 41.0 %, reflecting aged population in Japan. There were 170 deaths in the derivation cohort, resulting in in-hospital mortality of 2.0 %, and in 30-day mortality of 1.0 %, because a half of the death occurred later than 30 days. We identified four independent preoperative risk factors for in-hospital mortality: high-risk surgery [odds ratio (OR) 18.64], moderate-risk surgery (OR 5.00), ASA-PS ≥3 (OR 5.55), and emergency (OR 2.35). A good correlation between actual and calculated mortality based on the derivation cohort was confirmed in the validation cohort.

**Conclusions:**

This retrospective study of a single university hospital in Japan shows that aged patients in their 70 s is the largest group undergoing surgery, and that the overall in-hospital mortality is similar to that of other countries, but the 30-day mortality is less than that. Risk stratification for in-hospital mortality using preoperative factors was validated.

## Background

The number of elderly patients undergoing surgery has rapidly increased in Japan, which has one of the longest life expectancies in the world (Reich and Shibuya 2015). Elderly patients often have multiple co-morbidities and tend to suffer from major complications, even death, peri-operatively (Rockwood et al. 2005). However, a steady worldwide decline in peri-operative and anesthesia-related mortality over the last 50 years has been reported (Bainbridge et al. 2012; Semel et al. 2012). Advances in surgical and anesthetic technique and improved peri-operative care are thought to have contributed to the decline (Bainbridge et al. 2012). However, we do not have a clear idea of the outcome of current surgical procedures, especially in a tertiary hospital in Japan (Reich and Shibuya 2015). Thus, we aimed to gain an overview of recent surgical outcomes based on the mortality rate, its pre-operative risk factors, and the pattern of death using the administrative database of a tertiary university hospital.

Knowledge about overall outcomes is also important for patients and physicians. Patients have the right to be informed about the risks and benefits of surgical procedures based on the most recent data before making decisions on undergoing surgery. Hospitals can also use these data to benchmark its performance and improve the quality of care and efficacy.

## Methods

This retrospective cohort study protocol was reviewed and approved by the Kawasaki Medical School ethics committee with a waiver for individual informed consent (No. 1910). The hospital has a daily average of 650 in-patients and an emergency service with all specialties except transplantation surgery operating 24 h a day, 7 days a week. The hospital has 13 operating rooms and a postoperative intensive care unit (ICU) with 8 beds. We surveyed the electronic hospital records of in-patients who underwent surgery under general or neuroaxial nerve block by anesthesiologists from January 2010 through March 2014 at Kawasaki Medical School Hospital, a tertiary academic hospital in Okayama Prefecture, Japan. Surgeries performed under local anesthesia, such as ophthalmic and plastic surgeries, were not included. We also used the department’s anesthesia database to obtain additional data related to anesthesia. The two databases were downloaded into Microsoft Office Excel 2013 and combined for further statistical analysis.

### Patient variables, outcome measures and its prediction

Data included patient demographics, such as age, gender, weight and height; surgical/anesthesia data such as American Society of Anesthesiologists (ASA) physical status (PS), emergency (E) status, operative time, anesthesia time, surgical blood loss and presence of emergency repeat surgery during the same hospital stay; and clinical outcome measures consisting of in-hospital mortality and length of hospital stay. ASA-PS (2014) was classified by the attending anesthesiologists, and E was defined as surgery that was not scheduled on the daily surgical program and the necessity for surgery and anesthesia on that day that had been accepted by the supervising anesthesiologists.

The primary outcome was all-cause in-hospital mortality after surgery. Mortality was defined as death occurring after the first surgery during the same hospital stay. We selected in-hospital mortality because patients who may die of operative or peri-operative complications can do so after an arbitrary time point, such as 30 days in our hospital, because ill patients are not discharged from a tertiary hospital or transferred to another hospital within 30 days after surgery. Cases of inpatient surgery/anesthesia performed in a period between January 2010 and December 2011 were assigned to the derivation cohort to analyze the surgical outcomes and to develop a preoperative risk stratification formula. The reliability of the formula was tested in the validation cohort consisting of cases performed later than the period.

### Statistical analysis

Data were analyzed using Microsoft Excel for descriptive statistics. The incidence of mortality and other variables was assessed based on the first surgical case in each admission rather than per patient. First, two groups, patients with and without in-hospital mortality in the derivation cohort, were compared with respect to all demographic variables in univariate analyses using the Chi squared test for categorical variables and unpaired t tests for continuous variables. Second, the combined dataset was uploaded from Excel into SPSS version 22.0 (IBM). We performed logistic regression analysis with pre-operative variables using significant differences between two groups to identify pre-operative risk factors associated with in-hospital mortality. Descriptive data were presented as mean ± standard deviation (SD).

## Results

### Surgical patients

**Fig. 1:**
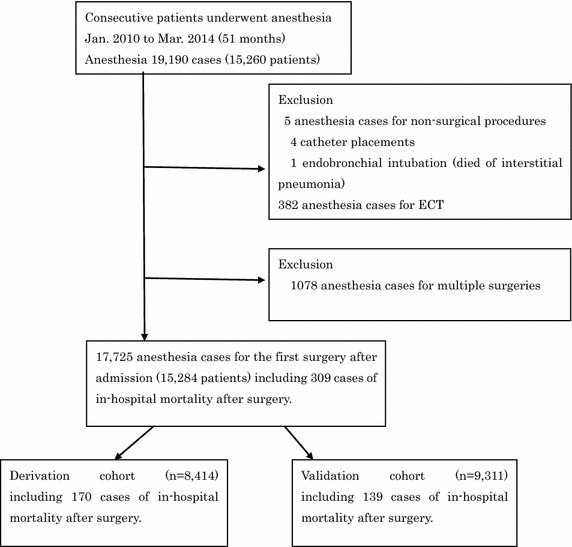
Flow diagram detailing the patients included in the retrospective analysis. Anesthesia cases for non-surgical procedures, such as central venous catheter and endobronchial double lumen tube placements, and electroconvulsive therapy (ECT) were excluded. Only the first surgery/anesthesia was included in the analysis when multiple procedures were performed during the same hospital stay. Surgery/anesthesia cases were divided into the derivation cohort and the validation cohort. Demographic data, pattern of in-hospital death and logistic regression analysis to define preoperative risk factors for the postoperative mortality were obtained using the former cohort. The latter served to validation of the preoperative risk stratification formula

**Fig. 2:**
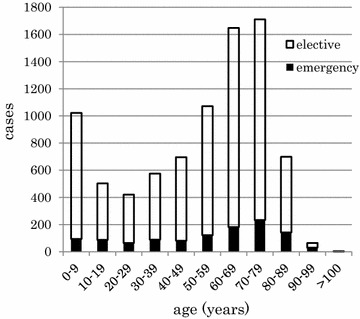
Age distribution of surgical patients who underwent surgical procedures between January 2010 and December 2011 at Kawasaki Medical School Hospital. The second or later surgery/anesthesia cases during one hospital stay were excluded. Among 8414 anesthesia/surgical cases, 7291 (86.7 %) cases were elective (*white bars*) and 1123 (13.3 %) cases were emergencies (*black bars*). The mean age was 52.0 ± 25.9 years with the largest age group in their 70 s. The percentage of patients aged ≥65 years was 41.0 %

During the period between January 2010 and April 2014, a total of 19,190 cases of inpatient surgery/anesthesia were performed in 15,260 patients at Kawasaki Medical School Hospital. Anesthesia for non-surgical procedures, such as central venous catheter (n = 5) and endobronchial double lumen tube (n = 1) placement, and electronic convulsive therapy (n = 382) were excluded from the analysis. We also excluded the second and subsequent surgical cases when multiple surgeries were performed during the same hospital stay. The hospital data based on the first surgery during each hospital admission was then divided into the derivation cohort consisting of 8414 anesthesia/surgery cases with 170 cases of in-hospital mortality, and the validation cohort of 9311 anesthesia/surgery cases with 139 cases of in-hospital mortality (Fig. 1). A histogram of the anesthesia/surgery cases according to patient age is shown in Fig. 2. The average patient age was 52.0 ± 25.9 years, with the largest age group in their 70 s. The percentage of patients aged ≥65 years was 41.0 %. The rate of emergency cases among all patients was 13.0 % and did not differ substantially among age groups.

The patient demographic and clinical characteristics of the non-dead and in-hospital mortality patients in the derivation cohort are compared in Table 1. Relevant intra-operative and postoperative outcome data are summarized in Table 2. Univariate analyses revealed significant differences in age and percentage of ≥75 years, gender, height, body mass index (BMI), ASA-PS, percentage of emergency surgery, percentage of emergency repeat surgery, surgery and anesthesia duration and percentage of blood loss >500 mL between the non-dead and in-hospital mortality patients in the derivation cohort (Tables 1, 2). Patients with in-hospital mortality stayed longer after surgery than other patients (Table 2). The crude mortality varied from 0 to 20.5 % among the types of surgery according to the surgical departments (Table 5 in Appendix). We classified surgical procedures with crude mortality greater >1 % as a high risk surgical group (highR; catheter intervention, emergency medicine, cardiovascular surgery, neurosurgery, gastrointestinal surgery, etc.), those with crude mortality of 0.5–1 % as a moderate risk surgical group (moderateR; orthopedics and respiratory surgery), and those with crude mortality <0.5 % as a low risk surgical group (lowR; Gynecology and obstetrics, urology, pediatric surgery etc) (Table 5 in Appendix).

**Table 1:** Baseline characteristics and in-hospital mortality in the derivation cohort

Characteristic	Non-dead n = 8244	In-hospital mortality n = 170	p value
Age (years)	51.6 ± 26.0	69.5 ± 14.6	<0.00001
≥75 years	1736 (21.1)	71 (41.8)	<0.00001
Female gender	3999 (48.5)	62 (36.5)	<0.0001
Height	152 ± 23.2	158.4 ± 11.2	<0.0003
Weight	52.5 ± 17.9	53.6 ± 13.0	0.2257
BMI	21.8 ± 4.4	21.2 ± 4.1	0.0393
ASA-PS			
ASA-I	3465 (42.0)	2 (1.2)	
ASA-II	3782 (45.9)	52 (30.6)	
ASA-III	859 (10.4)	74 (43.5)	
ASA-IV	136 (1.6)	38 (22.4)	
ASA-V	2 (0.0)	4 (2.4)	
ASA ≥ 3	997 (12.1)	116 (68.2)	<0.00001
Emergency surgery	1029 (12.5)	94 (55.3)	<0.00001

Data are given as the mean ± SD or n (%). Because some patients underwent more than one surgery/anesthesia during the same hospital stay, the analysis was made using the first surgery/anesthesia. In the database, height was not given for 17 (0.10 %) cases, weight for 11 (0.06 %) cases, and BMI for 19 (0.11 %) cases

**Table 2:** Comparison of intra-operative and postoperative outcomes in the non-dead and in-hospital mortality patients in the derivation cohort

Characteristic	Non-dead n = 8244	In-hospital mortality n = 170	p value
Surgery duration (min)	137 ± 113	186 ± 166	<0.00001
Anesthesia duration (min)	189 ± 118	242 ± 181	<0.00001
Blood loss (>500 mL)	594 (7.2)	52 (30.5)	<0.00001
Emergency repeat surgery	152 (1.8)	32 (18.8)	<0.00001
Hospital stay after surgery (days)	18.1 ± 27.1	44.0 ± 46.8	<0.00001

Data are given as the mean ± SD or n (%)

### Pre-operative risk factors associated with in-hospital mortality

Based on the results of univariate analyses of the pre-operative characteristics of the non-dead and in-hospital mortality patients in the derivation cohort we constructed a logistic regression model. The pre-operative risk factors for in-hospital mortality were HighR (odds ratio (OR) 18.64), ModerateR (OR 1.61) ASA-PS ≥ 3 (OR 5.55) and emergency surgery (OR 2.35) (OR 1.34; Table 3).

**Table 3:** Pre-operative risk factors for in-hospital mortality in the derivation cohort

Covariate	β	SE	p value	OR (95 % CI)
E	0.856	0.174	0.000	2.35 (1.67–3.31)
ASA ≥ 3	1.713	0.183	0.000	5.55 (3.87–7.95)
highR	2.93	0.468	0.000	18.64 (7.45–46.6)
moderateR	1.61	0.512	0.002	5.00 (1.83–13.62)

See “Appendix” for details of highR and moderateR

*ASA-PS* American Society of Anesthesiologists physical status, *E* emergency surgery, *OR* odds ratio, *SE* standard error, *CI* confidence interval, *highR* high risk surgical group, *moderateR* moderate surgical risk group

The probability of in-hospital mortality (P) is thus expressed based on the results of the logistic regression model as follows:

1 $$ {\text{P}} = \frac{\text{A}}{{1 + {\text{A}}}} $$

2 $$ {\text{A}} = \exp ( - 6.911 + 2.93 \times {\text{highR}} + 1.713 \times {\text{ASA-PS}} \ge 3 + 0.856 \times {\text{emergency}} + 1.61 \times {\text{moderateR}}) $$

We tested the validity of the formulas (Eqs. 1, 2) to predict postoperative in-hospital mortality in the validation cohort. The actual mortality and predicted mortality calculated using the probability equations were shown in Table 4. The comparison of the 12 paired risk stratification groups indicated a fairly good diagnostic performance of the formula based on these preoperative risk factor stratication. The calculated in-hospital mortalities from the derivation cohort for the highest risk groups in which patients have all of the pre-operative risk factors, i.e., emergency, high risk surgery and ASA ≥ 3, and the lowest risk groups in which they do not have any of those pre-operative risk factors were 19.5 and 0.1 %, respectively, whereas the actual values were 17.5 and 0.1 % in the validation cohort, respectively (Table 4).

**Table 4:** Actual and predicted mortality in the validation cohort

Emergency/elective	ASA-PS score	Risk of surgery	Cases (n)	Death (n)	Actual mortality (%)	Predicted mortality [% (95 % CIs)]
Elective						
	ASA ≦ 2	lowR	3756	3	0.1	0.1 (0.1–0.1)
		moderateR	2025	6	0.3	0.5 (0.2–1.3)
		highR	1610	24	1.5	1.8 (1.8–4.4)
	ASA ≥ 3	lowR	176	6	3.4	0.5 (0.4–0.8)
		moderateR	162	5	3.1	2.7 (0.7–9.7)
		highR	375	21	5.6	9.3 (6.7–27.0)
Emergency						
	ASA ≦ 2	lowR	175	1	0.6	0.2 (0.2–0.3)
		moderateR	192	0	0.0	1.2 (0.3–4.3)
		highR	466	12	2.6	4.2 (3.0–13.3)
	ASA ≥ 3	lowR	23	1	4.3	1.3 (0.6–2.6)
		moderateR	26	3	11.5	6.1 (1.2–26.3)
		highR	325	57	17.5	19.5 (10.7–55.0)
Total cases			9311	139	1.5	1.8 (1.2–5.1)

Actual and predicted mortality was compared in the validation cohort consisting of 9311 cases with 139 cases of in-hospital mortality. Patients were divided into 12 risk stratification groups according to the risk factors and the actual mortality and predicted mortality calculated using the probability equation (Eqs. 1, 2). The highest risk patients, i.e., emergency with ASA-PS 3 and high risk surgery, have a predicted mortality of 19.5 % and actual mortality of 17.5 %

*ASA* *≦* *2* ASA-PS score is 1 or 2, *ASA* *≥* *3* ASA-PS is 3 or greater than 3, *lowR* the low risk surgical group, *moderateR* the intermediate risk surgical group, *highR* the high risk surgical group

### Pattern of peri-operative deaths

**Fig. 3:**
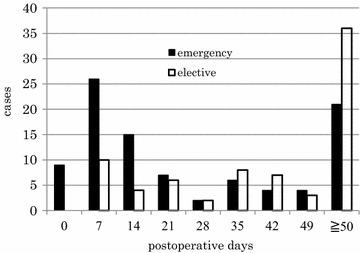
Distribution of postoperative days in patients who died in the hospital. A total of 170 patients (elective cases: 76, emergency cases: 94) died postoperatively during the hospital stay (44.0 ± 46.8 days). The mean postoperative days were significantly greater with elective surgery (59.2 ± 46.9 days) than emergency surgery (31.7 ± 43.2 days; p < 0.0001)

Postoperative hospital days after emergency and elective surgery are illustrated in Fig. 3. Death occurred at average 44.0 ± 46.8 days after surgery. Postoperative days to death were significantly greater after elective surgery (59.2 ± 46.9 days) than emergency surgery (31.7 ± 43.2 days). Furthermore, no patients died within 24 h after elective surgery, but 9 patients died within 24 h after emergency surgery. A total of 54 (71 %) and 35 (35 %) deaths occurring after elective surgery and emergency surgery, respectively, occurred later than 28 days after surgery. Thus, the 30-day mortality which is defined as death within 30 days after surgery was 1.0 %.

## Discussion

Minimizing peri-operative mortality and morbidity is an important and challenging task for surgical care teams. We found in this retrospective cohort study that 41.0 % of all surgical patients were ≥65 years old at a tertiary academic university hospital, reflecting our aging population in Japan, and that the in-hospital mortality was 2.0 % among 8414 surgeries and a half the in-hospital deaths occurred later than 30 days postoperatively, resulting in the 30-day mortality of 1.0 %. This study also demonstrated that certain types of surgery, such as cardiovascular, gastrointestinal, and neurosurgery, ASA-PS ≥ 3 and emergency surgery are the pre-operative risk factors predicting in-hospital mortality. Logistic regression analysis revealed that the probability of in-hospital mortality ranges between 0.1 and 19.5 % according to the pre-operative risks.

Although there are differences in study design, patient age and characteristics, and surgical procedures, the in-hospital mortality at our hospital is similar to the 30-day mortality in the nationwide study in the US. The 30-day mortality was reported to have declined nationwide from 1.68 % in 1996 to 1.32 % in 2006 despite the increasing age of surgical patients (Semel et al. 2012). Furthermore, the mortality in this study was significantly less than that at an American university-based tertiary hospital (Fecho et al. 2008), which reported a 30-day mortality of 2.1 %. However, there are still possibilities of reducing deaths from preventable errors (Bainbridge et al. 2012; Semel et al. 2012). One important approach is the enhanced adoption of evidence-based practices for peri-operative care. A two-fold variation in postoperative mortality has also been reported among hospitals in the United States, and the difference is attributed not to the rate of overall complications itself, but to the rate of rescue after major complications (Ghaferi et al. 2009). In accordance with this finding, Devereaux and Sessler (2015) suggested that intensifying postoperative monitoring and rapid management of cardiac complications is needed. We did not analyze the causes of in-hospital mortality because we do not think it is possible to clarify them in a retrospective study using administrative data. The nature of this retrospective study does not provide any evidence of causality for peri-operative mortality. Because we could not analyze the causes of deaths in our study, how many deaths we could prevent is debatable. We speculate, however, that a significant number of these patients suffered from major complications related to surgical procedures or postoperative care that do not allow them to be discharged from the hospital, as shown with significantly longer hospital stay and the greater percentage of emergency repeat surgery in the in-hospital mortality group. We need to better stratify cases by risk before surgery and re-consider indications for surgery in such patients in addition to greater postoperative care or vigilance in the surgical wards.

Whitlock et al. (2015) analyzed the national anesthesia clinical outcomes tegistry for early peri-operative mortality (within 48 h of inducing anesthesia) for the same time period as our study. They found 944 deaths (crude mortality rate, 0.03 %) among 2,866,141 cases. Despite differences in the definition of in-hospital mortality and the early mortality being 1/52 of our in-hospital mortality without time limit, the independent risk factors of early peri-operative mortality identified in that study were similar to the risk factors we identified. Therefore, these pre-operative risk factors play an important role in both early and late in-hospital mortality. Although beginning cases between 4:00 p.m. and 6:59 a.m. was pointed out as a preventable risk for early peri-operative mortality in their study, we do not have any data on the relationship between case start time and the in-hospital mortality in the current study.

The ASA classification does not intend to be an operative risk predictor, though it is very simple and accepted worldwide as a patient risk assessment scheme in anesthesia. A strong association between ASA classification and peri-operative mortality has been demonstrated in many studies over the past few decades (Bainbridge et al. 2012; Menke et al. 1993); the mean age of the surgical patients and number of co-existing diseases increased during this same period. Although surgical severity and invasiveness impacts in-hospital mortality (Liebman et al. 2010), it was not assessed in this study because we do not have a system for surgical severity in Japan. Such a surgical severity scale is useful not only for improving the outcome-based stratification of surgical patients, but also for training surgeons and coding surgical operations (Bainbridge et al. 2012). On the other hand, a standardized surgical technique is not possible in all specialties at a university hospital with teaching and training purposes. Thus, we used surgical departments as surrogates for surgical severity or invasiveness and identified low (lowR), high (highR) and intermediate risk (moderateR) surgeries based on crude mortality rates. This classification has the advantage that it is easy to use and relatively independent of the surgeon’s technique.

### Limitations

Firstly, this is a retrospective study using the administrative data from a single institution and does not represent the average status of all Japanese tertiary university hospitals. The hospital provides specialized medical care in all divisions and its high quality was acknowledged by the Japan council for quality health care. We think that the results of this study reflect the average performance of 79 tertiary university hospitals in Japan. Actually, the mean length of hospital stays in our hospital (13.61 days) was similar with the average of those in 79 tertiary university hospitals (13.99 days) in 2013 (http://www.mhlw.go.jp/stf/shingi/0000056344.html). This study has the advantages of a minimum amount of missing data in the patient database compared to a large scale multicenter study. Secondly, the retrospective nature of the study cannot exclude the possibility that unobserved confounding variables may affect mortality after surgery. We did not include pre-operative individual co-morbidities, such as diabetes, hypertension, atrial fibrillation, or congestive heart failure, in this analysis. It is plausible that the ASA-PS may have simply reflected patient co-morbidity in this study. Thirdly, cases in which a patient was discharged after surgery and readmitted to our hospital or other hospitals for complications and died are not counted as peri-operative mortality. In the Japanese health care system, ill patients are rarely discharged from the tertiary hospital or transferred to another hospital, as shown by the relatively long hospital stay of 14 days compared to other industrialized countries.

In conclusion, this retrospective cohort study of a single university hospital in Japan showed that aged patients in their 70 s are the largest group undergoing surgery. We found an overall in-hospital mortality of 2.0 % with a wide range of mortal days after surgery, resulting in the 30-day mortality of 1.0 %, which is less than that of other industrialized countries. Logistic regression analysis identified certain surgical specialties, ASA-PS ≥ 3, emergency surgery as pre-operative risk factors for peri-operative in-hospital mortality. Comparative studies with other hospitals are needed to validate our results.

## Appendix

See Table 5.

**Table 5:** Classification of surgical departments according to their mortality rates in the derivation cohort

Surgical department	Number of patients	Number of deaths	Crude mortality (%)	Risk classification
Catheter intervention	122	25	20.5	highR
Emergency medicine	50	4	8.0	highR
Neurosurgery	375	24	6.4	highR
Cardiovascular surgery	533	29	5.4	highR
Gastrointestinal surgery	1388	66	4.8	highR
Orthopedic	1780	15	0.8	moderateR
Thoracic surgery	250	2	0.8	moderateR
Gynecology and obstetrics	251	1	0.4	lowR
Urology	466	1	0.2	lowR
Pediatric surgery	549	1	0.2	lowR
Breast and thyroid surgery	681	1	0.1	lowR
Ear, nose, throat	799	1	0.1	lowR
Plastic surgery	672	0	0.0	lowR
Oral surgery	328	0	0.0	lowR
Ophthalmology	83	0	0.0	lowR
Dermatology	53	0	0.0	lowR
Others	34	0	0.0	lowR
Total	8414	170	2.0	

Mortality >1.0 % was classified into the high risk surgical group, 1–0.5 % the intermediate risk surgical group, and <0.5 % the low risk surgical group. Catheter intervention includes Cardiology, Stroke, Interventional Radiology, and Hepatopancreatology. Although these catheter intervention procedures are usually performed under local anesthesia, they are performed under general anesthesia if the case is life-threatening

## References

[CR1] ASA PHYSICAL STATUS CLASSIFICATION SYSTEM (2014) Last approved by the ASA House of Delegates on October 15, 2014. http://www.asahq.org/resources/clinical-information/asa-physical-status-classification-system

[CR2] Bainbridge D, Martin J, Arango M, Cheng D, Evidence-based Peri-operative Clinical Outcomes Research (EPiCOR) Group (2012). Perioperative and anaesthetic-related mortality in developed and developing countries: a systematic review and meta-analysis. Lancet.

[CR3] Devereaux PJ, Sessler DI (2015). Cardiac complications in patients undergoing major noncardiac surgery. N Engl J Med.

[CR4] Fecho K, Lunney AT, Boysen PG, Rock P, Norfleet EA (2008). Postoperative mortality after inpatient surgery: incidence and risk factors. Ther Clin Risk Manag.

[CR5] Ghaferi AA, Birkmeyer JD, Dimick JB (2009). Variation in hospital mortality associated with inpatient surgery. N Engl J Med.

[CR6] http://www.mhlw.go.jp/stf/shingi/0000056344.html

[CR7] Liebman B, Strating RP, van Wieringen W, Mulder W, Oomen JL, Engel AF (2010). Risk modelling of outcome after general and trauma surgery (the IRIS score). Br J Surg.

[CR8] Menke H, Klein A, John KD, Junginger T (1993). Predictive value of ASA classification for the assessment of the perioperative risk. Int Surg.

[CR9] Reich MR, Shibuya K (2015). The future of Japan’s health system-sustaining good health with equity at low cost. N Engl J Med.

[CR10] Rockwood K, Song X, MacKnight C, Bergman H, Hogan DB, McDowell I, Mitnitski A (2005). A global clinical measure of fitness and frailty in elderly people. CMAJ.

[CR11] Semel ME, Lipsitz SR, Funk LM, Bader AM, Weiser TG, Gawande AA (2012). Rates and patterns of death after surgery in the United States, 1996 and 2006. Surgery.

[CR12] Whitlock EL, Feiner JR, Chen LL (2015). Perioperative mortality, 2010 to 2014: a retrospective cohort study using the national anesthesia clinical outcomes registry. Anesthesiology.

